# Efficacy of High-Voltage Pulsed Radiofrequency in Zoster-Associated Pain: A Meta-Analysis and Systematic Review

**DOI:** 10.1155/2023/8479293

**Published:** 2023-12-23

**Authors:** Yinghao Song, Ziheng Yu, Jingjing Guan, Haisheng Wu, Jinglang Zhang, Liu Qiaoling, Min Yuan, Xinzhi Cheng, Bingyu Ling

**Affiliations:** ^1^Department of Pain, Clinical Medical College of Yangzhou University, Northern Jiangsu People's Hospital, Yangzhou, Jiangsu, China; ^2^Department of Emergency, Clinical Medical College of Yangzhou University, Northern Jiangsu People's Hospital, Yangzhou, Jiangsu, China

## Abstract

**Background:**

Zoster-associated pain (ZAP) is often refractory to conventional treatments and can seriously affect patients' physical and mental health. High-voltage pulsed radio frequency (H-PRF) is a new method for treating ZAP with pulse voltages above 60 V or even up to 100 V. The purpose of this paper was to conduct a systematic review and meta-analysis to evaluate the efficacy of H-PRF in the management of ZAP.

**Methods:**

PubMed, Embase, and the Cochrane library were searched from their inception to June 2022 to identify controlled trials which evaluated the effectiveness of H-PRF compared with standard PRF and sham operations. The primary outcome was pain scores at different treatment times. The secondary outcomes included SF-36 scores, rescue analgesic dose, and side effects.

**Results:**

We reviewed 6 randomized controlled trials involving 428 patients. There was no significant difference between the H-PRF and standard PRF pain scores at 1 week after surgery and the sham operation group at 1 month. At 1, 3, and 6 months, the H-PRF group had better pain score than the standard PRF group, and at 3 months, the pain score was better than the sham operation group. The H-PRF group showed improvement in the SF-36 score, and there were no significant complications in the H-PRF group.

**Conclusions:**

H-PRF is an effective and safe treatment method that has better effects in relieving pain and improving the quality of life and physical and mental health. Although H-PRF provides pain relief rates comparable to those of the control group in the early stages, it remains the preferred and alternative treatment for relieving herpes zoster-related pain.

## 1. Introduction

Zoster-associated pain (ZAP) is a neuropathic pain characterized by hyperalgesia and paresthesia, including herpetic pain and postherpetic neuralgia (PHN). It is an intractable, persistent, severe pain along the innervated skin area that occurs due to herpes zoster, which is troublesome to treat [1, 2]. In the United States, the overall incidence rate of PHN was 57.5 cases per 100,000 person-years, and the annual growth rate is 3.1% [3]. The prevalence of herpes zoster (HZ) and PHN in China is 7.7% and 2.3%, respectively, and about 29.8% of HZ patients will develop PHN, whose incidence increases with age, especially in elderly patients over 70 years [4]. The acute phase of herpes zoster usually occurs within one month after the eruption. Postherpetic neuralgia (PHN) is a kind of neuralgia that lasts one month after the explosion [5, 6]. ZAP is characterized by acupuncture, burning like an electric shock and severe pain, which leads to sleep disorders, anxiety, and depression and seriously affects patients' quality of life [7].

ZAP is a kind of refractory neuropathic pain. The commonly used treatment methods include oral drug therapy, nerve injection therapy, continuous epidural analgesia, etc., but these treatment methods are not effective enough for ZAP [8–11]. Pulsed radiofrequency (PRF) is an improvement to thermal radiofrequency therapy, in which a short (20 ms) burst of high-voltage current is followed by a silent phase (480 ms) to achieve heat transfer and maintain the target tissue temperature at 42°C. PRF technology is widely used in treating various neuropathic pains, especially in ZAP [12, 13]. Through image guidance, radiofrequency electrodes are punctured into the target nerve's dorsal root ganglion for treatment. The standard PRF (S-PRF) output voltage is 45 V, and its pain-relieving effect is controversial [14]. Some studies have shown that its analgesic effect is significant [15], but some believe its effect is limited and the recurrence rate is high [16]. Therefore, there is now a high-voltage pulsed radio frequency (H-PRF) modality that has been shown in some studies to provide better results [17]. Teixeira and Sluijter first reported that a high-voltage PRF ablation of 60 V used to treat patients with discogenic pain attained satisfactory efficacy that lasted over 3 months [18]. In 2013, Luo et al. found that there was a significant negative correlation between postoperative Numerical Rating Scale (NRS) scores and the output voltage of PRF in the treatment of idiopathic trigeminal neuralgia [19]. Subsequently, more and more studies have been conducted to treat ZAP by increasing the voltage value of pulsed radio frequency [20–22]. A randomized controlled trial (RCT) conducted by Wan et al. showed that no significant difference in improvement in pain scores was observed 1 month after treatment, and at 3 and 6 months, the high voltage group scored significantly lower than the standard voltage group; however, the incidence of ecchymosis in the high voltage group (19.2%) was higher than in the standard voltage group (12.1%) [23]. Therefore, further analysis is needed to determine whether H-PRF ablation is better than S-PRF ablation at different time points and whether H-PRF ablation is a safe treatment for ZAP. The purpose of this study was to compare the effectiveness of H-PRF in controlling ZAP, in order to better guide clinical work and improve the treatment effect of herpes zoster.

## 2. Materials and Methods

### 2.1. Data Sources and Search Strategies

Two researchers independently conducted comprehensive searches of PubMed, Embase, and the Cochrane library from their inception to June 2022. We used the following medical subject terms and keywords for our search: “postherpetic neuralgia,” “PHN,” “herpes zoster,” “herpetic neuralgia,” “zoster-associated neuralgia,” “pulsed radiofrequency,” “PRF,” “High-Voltage,” and “Voltage.” We use the “related articles” function in PubMed to expand the search scope and summarize all retrieved abstracts, studies, and citations. There were no language restrictions in this search. The search strategy is in supplementary material (available here). We preregistered the protocol of the systematic review on PROSPERO (CRD42022348310). Abbreviations can be found in Table 1.

### 2.2. Inclusion and Exclusion Criteria

Studies that met the following selection criteria were included in the meta-analysis: (1) The study was a randomized controlled trial (RCT). (2) The study group included an H-PRF and a control group, while the control group included standard PRF and sham operation. (3) The study subjects were adults with acute herpes, subacut herpes, and PHN, collectively referred to as ZAP. (4) They reported at least one outcome indicator of the visual analog scale (VAS), Numerical Rating Scale (NRS), 36-Item Short-Form Health Survey (SF-36), rescue analgesic dose, and side effects.

The exclusion criteria were as follows: (1) patients with sequelae of cerebral thrombosis, severe cardiopulmonary disease, serious liver and kidney dysfunction, and other serious systemic diseases; (2) reviews, animal studies, and case reports; (3) the data from the study were not suitable for statistical analysis; and (4) patients with other neuropathies or neurological diseases (e.g., diabetes mellitus neuropathy, intercostal neuralgia, and primary trigeminal neuralgia).

### 2.3. Data Extraction and Quality Assessment

Two reviewers independently extracted baseline data and result data. We extracted the following data from the included studies: the name of the first or corresponding author, the year of publication, the type of study, the sample size, the last follow-up time, the age and sex of the study population, the location of herpes, RF parameters, and outcome indicators. The quality of RCTs was assessed using the bias risk assessment tool of Cochrane Collaboration. The judgments of bias were reported as “low risk,” “high risk,” or “unclear risk” according to their methodological section.

### 2.4. Statistical Analysis

This study used RevMan5.3 (Nordic Cochrane Collaborative Centre Cochrane, Copenhagen, Denmark) for meta-analysis. We used weighted mean differences and their 95% confidence intervals for analysis for continuous variables. A *P* value <0.05 was assigned statistical significance, and a *P* value <0.01 was given significant statistical significance [24, 25]. *I*^2^ test was used to test the heterogeneity between studies. The random effect model is used in this study. Subgroup analysis was used to assess heterogeneity. If there were only graphs in the literature, two researchers independently used the GetData Graph Digitizer 2.25 to extract the data and calculate the mean and standard deviation [26, 27].

## 3. Results

### 3.1. Study Selection

**Figure 1:**
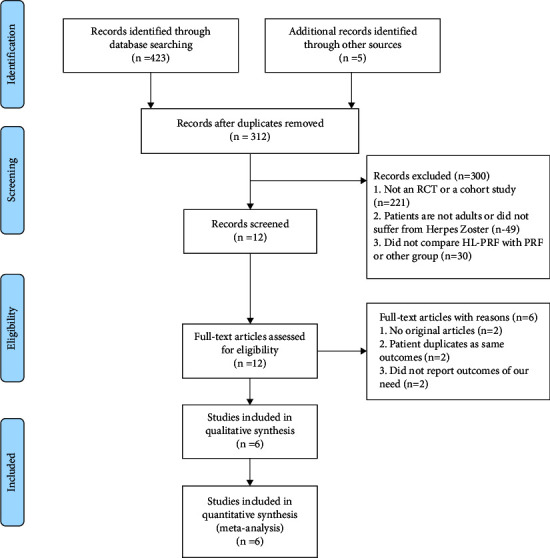
Flowchart illustrating the study selection process.

Figure 1 is a flowchart depicting the screening and selection of studies. A search of this database yielded 428 articles. After reviewing the titles and abstracts, 416 articles were excluded. Finally, six full-text articles that met our requirements were retrieved.

### 3.2. Study Characteristics

Our meta-analysis included 456 patients (227 received H-PRF, 152 received S-PRF, and 77 received sham surgery). All participants were diagnosed with ZAP and received H-PRF or other treatment. Three trials [20, 22, 28] included patients who had suffered ZAP within 1–3 months. Two trials [14, 23] included patients who had suffered ZAP within 1 month, while one trial [29] involved patients who had suffered ZAP for around 3 months. The herpes sites in three trials [20, 22, 28] were the trigeminal nerve, two in the thoracic back [23, 29], and one in the neck and upper extremities [14]. Before and after treatment, all included trials [14, 20, 22, 23, 28, 29] reported pain scores to assess pain intensity. Four trials [20, 22, 28, 29] reported SF-36 while others [14, 23] reported PSQI or SQS. Four trials reported changes in drug dosage [20, 22, 23, 28], and five trials reported adverse events after treatment [14, 20, 22, 28, 29]. Follow-up lasted for 2 days to 12 months in the included studies. The characteristics of this study are shown in Table 2.

### 3.3. Risk of Bias Assessment

**Figure 2:**
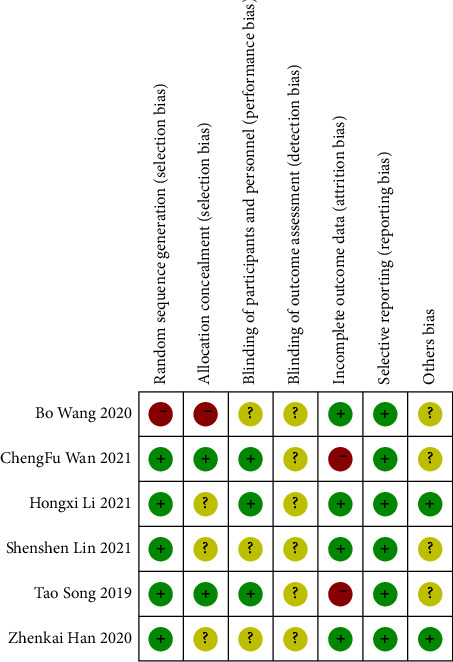
Risk of bias assessment using the cochrane risk-of-bias tool for randomized controlled trials included in a meta-analysis.

Five trials [14, 20, 22, 28, 29] were assessed as “low risk” for sequence generation because participants were grouped by a numerical randomisation method. One trial [23] was classified as “high risk” due to the lack of reporting of appropriate random sequence generation methods and the lack of assignment concealment. Because of the lack of information, three trials [14, 28, 29] were deemed to be concealing the assigned “undefined risk.” Two trials [20, 22] were rated as “low risk” for the project because their allocation procedures depended on computer-generated allocation sequences. Three trials [14, 23, 29] showed performance bias due to a lack of blind information about people and outcome evaluation. Three trials [20, 22, 28] reported that the trial was classified as “low risk” of performance bias due to the carefully designed S-PRF group and sham surgery group, with staff and participants unaware of the grouping. Due to the lack of information, detection bias was assessed as an “undefined risk” in all trials. Five patients in two trials [20, 22] lost follow-up without explanation, which may have resulted in incomplete bias in the outcome data. Other trials were considered “low risk” because no incomplete outcome data was detected. None of the included trials showed selective reporting bias, but four of them [14, 20, 22, 23] did not report conflicts of interest and funding sources, which could have contributed to other biases. As no more than 10 studies were included, publication bias was not assessed. The quality evaluation of randomized controlled trials is shown in Figure 2.

### 3.4. Pain Scores

#### 3.4.1. H-PRF and S-PRF

**Figure 3:**
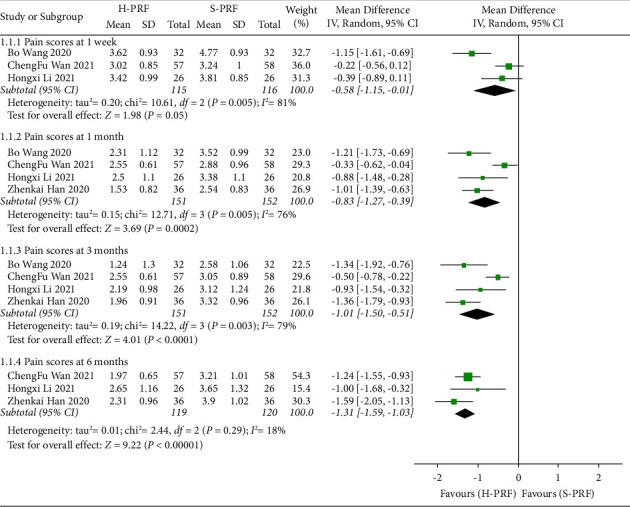
Forest plot of pain scores for H-PRF and S-PRF at one week, one month, three months, and six months after operation in a meta-analysis.

The subgroup analysis of 1 week [22, 23, 28] after intervention showed that both H-PRF and S-PRF had a similar effect on relieving pain intensity (*P*=0.05; weighted mean difference (WMD), −0.58; 95% CI, −1.15 to −0.01; *I*^2^ = 81%). However, subgroup analysis of 1 month [22, 23, 28, 29], 3 months [22, 23, 28, 29], and 6 months [22, 23, 28, 29] indicated that H-PRF was more effective in pain reduction than S-PRF (month 1: *P* < 0.01; WMD, −0.83; 95%C I, −1.27 to −0.39; *I*^2^ = 76%; month 3: *P* < 0.01; WMD, −1.01; 95% CI, −1.50 to −0.51; *I*^2^ = 79%; month 6: *P* < 0.01; WMD, −1.31; 95% CI, −1.59 to −1.03; *I*^2^ = 18%), as shown in Figure 3.

#### 3.4.2. H-PRF and Sham

**Figure 4:**
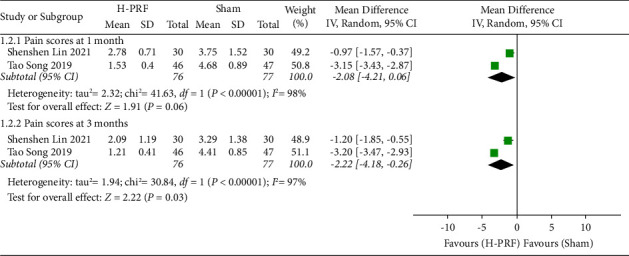
Forest plot of pain scores for the H-PRF and sham group at one month and three months after operation in a meta-analysis.

Two studies [14, 20] compared the pain scores at different times after H-PRF and sham treatment, both of which used VAS scores, and were grouped and analyzed according to 1 month after treatment and three months after treatment. Two studies [14, 20] showed no statistically significant in the H-PRF group and the sham treatment group at 1 month (*P*=0.06; WMD, −2.08; 95% CI, −4.21 to −0.06; *I*^2^ = 97%). Two studies [14, 20] showed that the H-PRF group had lower pain scores at 3 months after treatment than the sham treatment group (*P*=0.03; WMD, −2.22; 95% CI, −4.18 to −0.26; *I*^2^ = 97%), as shown in Figure 4.

### 3.5. 36-Item Short Form Health Survey

The SF-36 score of the last follow-up was included in this study. The follow-up time was at least three months. The SF-36 score had bodily pain, general health, mental health, physical function, role-emotional, social function, and vitality.

#### 3.5.1. H-PRF and S-PRF

**Figure 5:**
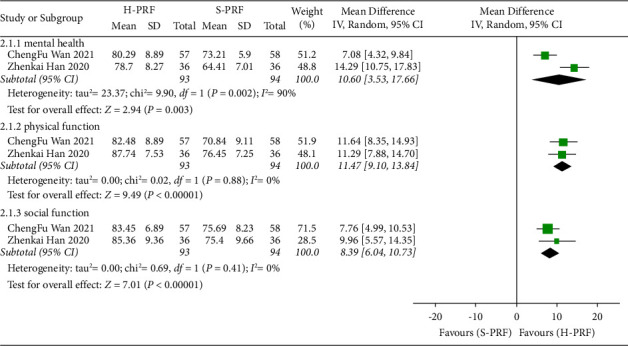
Forest plot of SF-36 score for H-PRF and S-PRF at the last follow-up time after operation in a meta-analysis.

Two studies [22, 29] compared H-PRF and S-PRF in SF-36 score. Compared with the S-PRF group, H-PRF treatment significantly improved the mental health score (*P* < 0.01; WMD, 10.6; 95% CI, 3.53 to 17.66; *I*^2^ = 90%), physical function score (*P* < 0.01; WMD, 11.47; 95% CI, 9.10 to 13.84; *I*^2^ = 0%), and social function score (*P* < 0.01; WMD, 8.39; 95% CI, 6.04 to 10.73; *I*^2^ = 0%) in SF-36, as shown in Figure 5.

#### 3.5.2. H-PRF and Sham

**Figure 6:**
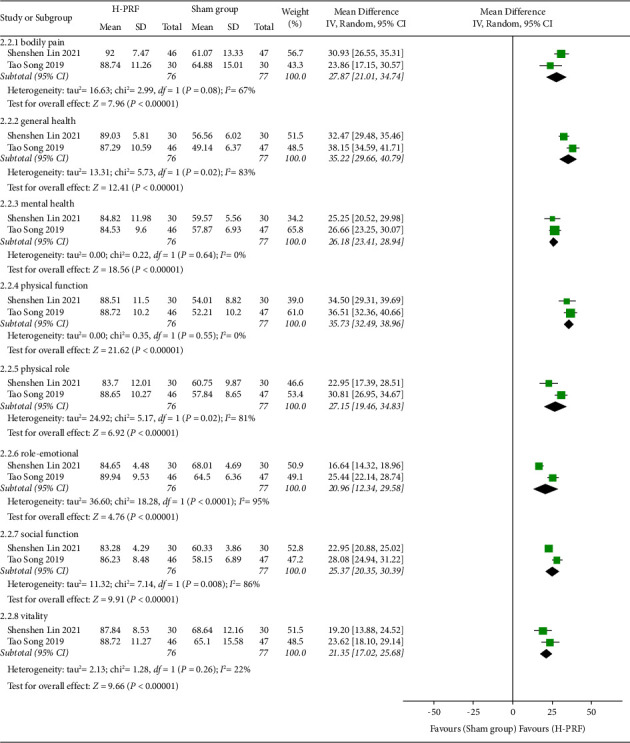
Forest plot of SF-36 score for the H-PRF and sham group at the last follow-up time after operation in a meta-analysis.

In the two studies [14, 20], the H-PRF group was significantly higher than the sham treatment group in bodily pain (*P* < 0.01; WMD, 27.87; 95% CI, 21.01 to 34.74; *I*^2^ = 67%), general health (*P* < 0.01; WMD, 35.22; 95% CI, 29.66 to 40.79; *I*^2^ = 83%), mental health (*P* < 0.01; WMD, 26.18; 95% CI, 23.41 to 28.94; *I*^2^ = 0%), physical function (*P* < 0.01; WMD, 35.73; 95% CI, 32.49 to 38.96; *I*^2^ = 0%), physical role (*P* < 0.01; WMD, 27.15; 95% CI, 19.46 to 34.83; *I*^2^ = 81%), role-emotional (*P* < 0.01; WMD, 20.96; 95% CI, 12.34 to 29.58; *I*^2^ = 95%), social function (*P* < 0.01; WMD, 25.37; 95% CI, 20.35 to 30.39; *I*^2^ = 86%), and vitality (*P* < 0.01; WMD, 21.35; 95% CI, 17.02 to 25.68; *I*^2^ = 22%) of SF-36, as shown in Figure 6.

### 3.6. Rescue Analgesic Dose

Four randomized controlled trials [14, 20, 22, 23] reported the effect of pain control by recording the dose of analgesics. Due to the differences in the category of analgesic drugs, data format, data collection time, and unit in the included randomized controlled trials, we did not have this result in the meta-analysis but only made a descriptive analysis. Wan et al. [22] and Wan et al. [20] reported pregabalin dosage in the H-PRF group compared with the control group. Wang et al. [23] and Lin et al. [14] reported the dosage of gabapentin and tramadol. Due to the difference between the time and unit of data collection, these data cannot be summarized. All four randomized controlled trials [14, 20, 22, 23] showed that the analgesic dose of the H-PRF group was significantly lower than that of the standard PRF group and sham group.

### 3.7. Side Effects

All RCTs did not report serious adverse effects after H-PRF, such as intraspinal/paravertebral hematoma, hemorrhage, infection, hoarseness/aphonia, pneumothorax hypoesthesia of face or weakness of masseter muscle, and intracranial hemorrhage.

#### 3.7.1. H-PRF and S-PRF

**Figure 7:**
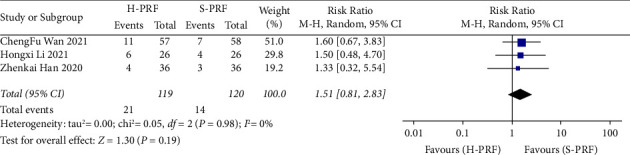
Forest plot of side effects for H-PRF and S-PRF in a meta-analysis.

Three studies [22, 28, 29] compared the side effects of H-PRF and S-PRF, including ecchymosis, bradycardia, tachycardia, local swelling, worsened pain, dizziness, nausea, and vomiting. There was no statistically significant difference in the occurrence of these side effects (*P*=0.19; RR, 1.51; 95% CI, 0.81 to 2.83; *I*^2^ = 0%), as shown in Figure 7.

#### 3.7.2. H-PRF and Sham

**Figure 8:**
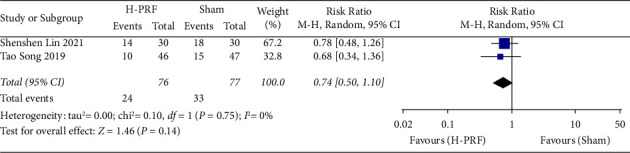
Forest plot of side effects for the H-PRF and sham group in a meta-analysis.

Two studies [14, 20] compared the side effects of H-PRF and sham treatment, including ecchymosis, tachycardia, nausea, vomiting, and hypertension. There was no statistically significant difference in the occurrence of these side effects (*P*=0.13; RR, 0.74; 95% CI, 0.50 to 1.10; *I*^2^ = 0%), as shown in Figure 8.

### 3.8. Sensitivity Analysis

All included studies were excluded one by one, and the results showed no significant changes in heterogeneity between the studies, while the meta-analysis results remained consistent.

## 4. Discussion

There are three main findings in our meta-analysis. First, H-PRF significantly alleviated ZAP when compared with the control group, but the advantages are not so obvious in the early stages. Second, H-PRF seemed to better improve the quality of life and physical and mental health of patients. Third, no serious adverse events were observed, and the reported complications were comparable between the two groups.

ZAP is refractory neuralgia, and no standard treatment algorithm is devised for all ZAP patients [30]. According to the search results, there is no meta-analysis of the efficacy of H-PRF compared with other treatment methods, so this study is the first. In this meta-analysis, we investigated the effectiveness of H-PRF in reducing pain, improving quality of life, and enhancing physical and emotional functioning in patients with HZ. According to the search, there was no difference in pain relief between H-PRF and S-PRF at 1 week. Compared with the sham operation group, there was no difference in pain relief at 1 month. However, the improvement of pain scores at 1, 3, and 6 months after operation, the H-PRF outperformed the S-PRF. The H-PRF group was better than the sham group in pain scores at 3 months after operation. In terms of pain improvement in the medium to long term, the H-PRF has more advantages. The results show that the analgesic effects of PRF appear to develop slowly and take longer to achieve optimal effects. At the same time, this finding is consistent with other literature suggesting that the neuroregulatory effects of PRF occur gradually and reach their maximum at 3 months after the intervention [31]. The meta-analysis by Wang et al. [32] also showed that the PRF group did not begin to outperform the control group in terms of pain reduction until after 1 week.

SF-36 is a practical and common tool for assessing health status and is often used to evaluate the quality of life of ZAP patients [21, 33, 34]. This meta-analysis showed that patients in the H-PRF group had higher mental health, physical function, and social function scores than in the S-PRF group. Compared with the sham group, the scores of all items in SF-36 in the H-PRF group were higher. The H-PRF group improved quality of life, which was associated with what we believe to be better Analgesia. H-PRF can provide ZAP patients with the advantage of better mental health and faster return to life and society. Although we cannot summarize and analyze the dosage of analgesic drugs, from a single study perspective, H-PRF can better reduce the dosage of drugs, which means that patients can reduce the impact of drug side effects. PRF will not destroy the anatomical basis of pain pulse transmission nor cause nerve injury and protein coagulation. Its main role is regulating nerve function [12, 31, 35, 36]. This study found that high-voltage pulse radiofrequency surgery will not cause numbness, nerve injury, or other symptoms, so the safety is as high as the pulse frequency. Compared to traditional thermocoagulation radiofrequency therapy, pulsed radiofrequency current is uninterrupted. This energy transfer does not disrupt the anatomical basis of pain pulse transmission, nor does it cause nerve damage and protein coagulation [29]. The pulse radio frequency has a silent stage and sufficient time to eliminate heat. Therefore, even if the voltage is increased, it still does not cause damage to the nerves.

In the acute phase of herpes zoster, the VZV-induced ganglions cause a strong local sympathetic response, leading to vasoconstriction, ischemic nerve damage, and pain [37]. Pathological changes in the acute phase of HZ can cause peripheral and central sensitization in time, which will downregulate the central pain inhibition pathway, change the gene expression encoding neuropeptides, and expand the receptive field [38]. Sensitization results in the hyperexcitability of dorsal horn neurons. These damaged sensory neurons produce abnormal electrical impulses that are transmitted to the spinal cord, inducing spontaneous pain, which is characterized by ectopic pain, hyperalgesia, burning, and electric shock-like symptoms [37–39]. Current studies have shown that PRF generates radiofrequency electric field effects at target tissues, modulates abnormally active synaptic conduction in chronic pain, and at the same time, changes nerve fiber structure [40], and affects peripheral nerve cell ion channels to improve ZAP peripheral sensitization. PRF can increase the expression of transcription activator-3 in C and A*δ* fibers of dorsal root ganglion pain conduction, thereby activating the descending inhibitory system of the brainstem, resulting in an analgesic effect [41].

PRF is a new therapeutic technique. Currently, how to maximize the therapeutic effect of PRF has always been a significant problem faced by clinicians and scientists. Nowadays, many basic experiments and clinical studies have been carried out on parameters such as target, time, waveform, temperature, voltage, etc. Currently, no international guidelines recommend an ideal PRF setting for treating neuropathic pain [42–44]. The standard RF mode has a frequency of 2 Hz, output voltage of 45 V, pulse width of 20 ms, and upper temperature limit of 42°C, but many clinical patients cannot achieve satisfactory curative effect [8–11]. Therefore, some scholars began to change the voltage parameters and found that good clinical efficacy could be achieved. In 2006, Teixeira et al. used a high voltage of 60 V for the first time in patients with lumbar discogenic pain and achieved satisfactory results [18]. It was believed that the therapeutic effect of PRF was due to the influence of the electric field effect generated by the electrode on the nerve rather than the thermal effect. In the study we included, there are two modes of high voltage: one is to increase from 40 V to the patient's maximum tolerance gradually, usually 60−100 v [14, 20, 22, 23], and the other is to increase progressively from 40 V to 65 V [28, 29], but keep the other parameters the same as the standard RF.

Our research has several limitations. First, the number of RCTs included is small, and the sample size is small. Secondly, considering the limited number of randomized controlled studies on high voltage, we are unable to classify herpes zoster-related neuralgia into herpetic neuralgia and postherpetic neuralgia for subgroup analysis. Third, the follow-up time included in the study is short, no more than half a year, so it is impossible to compare the long-term efficacy. Finally, the data extraction method (GetData graph digitizer) may limit the statistical power and accuracy.

## Figures and Tables

**Table 1:** Abbreviations used in the text.

Zoster-associated pain	ZAP
Randomized controlled trial	RCT
Visual analog	VAS
Postherpetic neuralgia	PHN
High-voltage pulsed radiofrequency	H-PRF
Standard pulsed radiofrequency	S-PRF
Numerical Rating Scale	NRS
36-Item Short Form Health Survey	SF-36
Sleep Quality Scale	SQS
The Pittsburgh Sleep Quality Index	PSQI
Day (d), week (w), month (m)	Pain duration

**Table 2:** The characteristics of the studies included in this meta-analysis.

Study	Level of study	Group	Sex (M/F)	Age, mean ± SD (y)	Pain duration, mean ± SD	Herpes involved area	Intervention (V)	Last follow-uptime (m)	Outcome
Cheng-Fu Wan, 2021	RCT	S-PRF	23/35	69.96 ± 13.66 d	65.14 ± 18.53 d	Trigeminal	40	6	VAS, SF-36 score, mean dose of pregabalin, adverse events
		H-PRF	21/36	70.54 ± 14.02	67.28 ± 19.64 d		60 V to 100		

Hongxi Li, 2021	RCT	S-PRF	10/16	64.15 ± 12.29	56.69 ± 13.70 d	Trigeminal	45	6	VAS, SF-36, treatment efficiency, adverse events
		H-PRF	12/14	66.62 ± 8.21	58.85 ± 16.62 d		65		

Tao Song, 2019	RCT	Sham group	20/27	65.96 ± 13.66	63.14 ± 18.53 d	Trigeminal		6	VAS, SF-36, dosage of pregabalin, adverse events
		H-PRF	21/25	65.54 ± 13.28	59.28 ± 16.64 d		60 to 90		

Bo Wang, 2020	RCT	S-PRF	13/19	71.42 ± 5.43	22.40 ± 5.46 d	Thoracolumbar	47.73 ± 2.45	3	NRS, SQS, gabapentin and tramadol doses, clinically meaningful PHN cases
		H-PRF	15/17	72.81 ± 5.92	23.20 ± 4.61 d		76.50 ± 5.61		

Zhenkai Han, 2020	RCT	S-PRF	17/19	67.67 ± 6.77	3.08 ± 1.07 m	Thoracolumbar	45	12	VAS, SF-36, patient satisfaction, adverse events
		H-PRF	16/20	68.19 ± 10.42	3.38 ± 0.93 m		65		

Shenshen Lin, 2021	RCT	Sham group	16/14	67.6 ± 10.6	16.0 ± 8.7 d	Neck, upper limbs, etc		3	VAS, PSQI, SF-36, use of tramadol and gabapentin, adverse events
		H-PRF	18/12	66.8 ± 8.4	15.2 ± 7.3 d		90 V–100		

## Supplementary Materials

Supplementary Materials The supplementary materials describe the search strategy.
