# Supplementary material for: Efficacy of High-Voltage Pulsed Radiofrequency in Zoster-Associated Pain: A Meta-Analysis and Systematic Review
Source: Anesthesiol Res Pract. 2023 Dec 23;2023:8479293. doi: 10.1155/2023/8479293 (PMC10757660; doi:10.1155/2023/8479293)
Supplement: Supplementary Materials — The supplementary materials describe the search strategy. [file 8479293.f1.docx]

Pumbed

#1 Herpes Zoster[Mesh]

#2 "Neuralgia, Postherpetic"[Mesh]

#3 #1 OR #2

#4 (postherpetic neuralgia[All Fields]) OR (PHN[All Fields]) OR (herpetic neuralgia[All Fields]])OR (zoster-related pain[All Fields]) OR (zoster-related neuralgia[All Fields])

#5 #3 OR #4

#6 "Pulsed Radiofrequency Treatment"[Mesh]

#7 (pulsed radiofrequency[All Fields]) OR (pulsed neuromodulatory[All Fields]) OR (PRF[All Fields]) OR (pulsed[All Fields]) OR (radiofrequency[All Fields])

#8 #6 OR #7

#9 Voltage[All Fields])

#10 High-Voltage[All Fields]

#11 #9 OR #10

#12 randomized controlled trial[Publication Type] OR randomized[Title/Abstract] OR placebo[Title/Abstract]

#13 #5 AND #8 AND #11 AND #12

Embase

#1. 'herpes zoster'/exp

#2. 'postherpetic neuralgia'/exp

#3. 'postherpetic neuralgia' OR phn OR 'herpetic

neuralgia' OR 'zoster-related pain' OR

'zoster-related neuralgia'

#4. #1 OR #2 OR #3

#5. 'pulsed radiofrequency treatment'/exp

#6. 'pulsed radiofrequency' OR 'pulsed

neuromodulatory' OR prf OR pulsed OR

radiofrequency

#7. #5 OR #6

#8. voltage OR 'high-voltage'

#9. #4 AND #7 AND #8

Cochrane Library

ID Search

#1 MeSH descriptor: [Herpes Zoster] explode all trees

#2 MeSH descriptor: [Neuralgia, Postherpetic] explode all trees

#3 #1 OR #2

#4 postherpetic neuralgia

#5 PHN

#6 herpetic neuralgia

#7 zoster-related pain

#8 zoster-related neuralgia

#9 #3 OR #4 OR #5 OR #6 OR #7 OR #8

#10 MeSH descriptor: [Pulsed Radiofrequency Treatment] explode all trees

#11 pulsed radiofrequency

#12 pulsed neuromodulatory

#13 PRF

#14 pulsed

#15 radiofrequency

#16 #10 OR #11 OR #12 OR #13 OR #14 OR #15

#17 Voltage

#18 High-Voltage

#19 #17 OR #18

#20 #9 AND #16 AND #19
